# Angiotensin-Converting Enzyme Inhibition and Parathyroid Hormone Secretion

**DOI:** 10.1155/2017/4138783

**Published:** 2017-07-20

**Authors:** Sarah Zaheer, Jenifer M. Brown, Molly Connors, Jonathan S. Williams, Gail K. Adler, Anand Vaidya

**Affiliations:** ^1^Division of Endocrinology, Diabetes, and Hypertension, Brigham and Women's Hospital, Harvard Medical School, Boston, MA, USA; ^2^Department of Medicine, Brigham and Women's Hospital, Harvard Medical School, Boston, MA, USA

## Abstract

**Background:**

Prior studies suggest that renin-angiotensin-aldosterone system (RAAS) inhibitors decrease parathyroid hormone (PTH) secretion.

**Objective:**

To evaluate the effect of angiotensin-converting enzyme inhibitors (ACEi) on serum PTH in participants with and without primary hyperparathyroidism (P-HPT).

**Methods:**

An open-label, single-arm, pilot study whereby participants with and without P-HPT had PTH were evaluated before and after 1 week of maximally tolerated lisinopril therapy.

**Results:**

A total of 12 participants with, and 15 participants without, P-HPT successfully completed the protocol. Following 1 week of lisinopril, participants with P-HPT had a decrease in systolic blood pressure (SBP) (−6.4 mmHg, *P* < 0.01), an increase in plasma renin activity (PRA) (+1.50 ng/mL/h, *P* = 0.06), and a decrease in PTH (79.5 (21.6) to 70.9 (19.6) pg/mL, ∆ = −8.6 pg/mL, *P* = 0.049); however, serum and urine calcium did not change. In contrast, although 1 week of lisinopril significantly decreased SBP and increased PRA among participants without P-HPT, there were no changes in PTH or calcium.

**Conclusion:**

In this short pilot investigation, 1 week of maximally titrated ACEi did not impact PTH in participants without P-HPT, but resulted in a modest and marginally significant reduction of PTH but not calcium, among participants with P-HPT. This trial is registered with ClinicalTrials.gov NCT01691781.

## 1. Introduction

Renin-angiotensin-aldosterone system (RAAS), the key hormonal regulator of sodium and volume homeostasis, also plays a major role in the pathogenesis of cardiovascular disease [1, 2]. Pharmacologic inhibition of the RAAS is a cornerstone of treatment for hypertension, coronary artery disease, and heart failure [3–5].

Parathyroid hormone (PTH) is a well-established regulator of calcium and skeletal homeostasis. Observational studies have shown that higher PTH levels are independently associated with higher blood pressure and increased risk for incident hypertension, cardiovascular mortality, and structural cardiac dysfunction [6–10]. Though several hypotheses have been proposed to explain these observations, there is compelling evidence that an interaction exists between the RAAS and PTH that may explain these observational findings [11–14].

Individuals with primary aldosteronism have higher PTH levels when compared to matched individuals with essential hypertension [13, 15–17]. Even in individuals without primary aldosteronism, higher serum aldosterone levels are independently associated with higher PTH levels [18]. We previously reported that infusion of angiotensin II in individuals without primary hyperparathyroidism (P-HPT) increased PTH levels by >30% [19]. Since we [19], and others [16], have shown that both the angiotensin type I receptor (ATR1) and the mineralocorticoid receptor (MR) are expressed in parathyroid tissue, we hypothesized that these findings may have been mediated by activation of the parathyroid AT1R and/or MR. Some studies suggest that this interaction may be modifiable: among individuals without P-HPT, ACE inhibitors and angiotensin receptor blockers have been associated with lower PTH [18, 19]. Further, studies in participants without P-HPT suggest that MR antagonists associate with lower PTH [19, 20]; however, a recent placebo-controlled and randomized clinical trial reported that MR antagonist therapy did not influence PTH levels in subjects with P-HPT [21].

Based on this accumulation of suggestive but also inconsistent evidence of a modifiable interaction between the RAAS and PTH, we conducted a single-arm, open-label pilot study to assess whether ACE inhibitors decrease PTH levels in patients with P-HPT. The impetus to evaluate the clinical applicability of ACE inhibitors in P-HPT is based on the hypothesis that they may have a dual benefit in lowering the risk for both adverse skeletal [22] and cardiovascular outcomes [23, 24].

## 2. Materials and Methods

### 2.1. Study Participants

Participants were recruited from the greater Boston area, and the trial was conducted at the Clinical Research Center at Brigham and Women's Hospital in Boston, Massachusetts. All participants provided informed consent, and the study protocol was approved and monitored by our institutional human research and ethics committee (NCT01691781). Participants with P-HPT were recruited from Endocrinology and Endocrine Surgery Clinics at Brigham and Women's Hospital and affiliated hospitals. Participants without P-HPT were recruited from healthy volunteers. Participants underwent a screening visit with a study physician to determine eligibility.

Participants with primary hyperparathyroidism were included if they had a biochemical diagnosis of P-HPT confirmed by their endocrinologist, were normotensive or had mild (stage I) hypertension that was untreated or treated with a single antihypertensive agent, were between the ages of 18 and 80 years, and had normal estimated glomerular filtration rate (eGFR > 60 mL/min/1.73m^2^). Participants without P-HPT were included based on the same inclusion criteria except they could not have a known diagnosis of P-HPT.

Exclusion criteria for all participants were presence of chronic kidney disease defined as eGFR <60 mL/min/1.73m^2^, stage 2 or 3 hypertension or use of >1 antihypertensive medication, type 2 diabetes not controlled by diet or metformin alone or A1c > 7.5%, history of liver or heart failure, use of antipsychotic medication or lithium, presence of chronic inflammatory condition treated with prescribed nonsteroidal anti-inflammatory drugs (NSAIDs), use of prescribed doses of potassium supplements, illness requiring overnight hospitalization in the last 6 months, or pregnancy/breast-feeding. Participants with P-HPT who were in the midst of planning a parathyroidectomy were also excluded.

The initial objective of this pilot study was to enroll participants over a project period of up to four years, or until a maximum of 15 participants without P-HPT, or 30 participants with P-HPT, were enrolled.

### 2.2. Antihypertensive Medication Washout Protocol

All enrolled participants who were on a single antihypertensive medication underwent a medication washout period before initiation of study procedures to avoid interference with RAAS and calcium-regulatory physiology. Angiotensin-converting enzyme inhibitors (ACE inhibitors), angiotensin receptor blockers, and mineralocorticoid receptor antagonists were stopped for 2 months, beta-blockers and diuretics were stopped for 1 month, and calcium-channel blockers were stopped for 2 weeks prior to the start of any study procedures. During the medication washout period, participants were given home sphygmomanometers to measure their daily blood pressure at home and report blood pressure readings to study staff. If blood pressures exceeded 159/99 mmHg for more than 1 week, a study physician considered either withdrawing the participant from the study or initiating amlodipine to lower blood pressure during the washout period. If amlodipine was started, it was discontinued 2 weeks prior to starting the study procedures. Participants whose blood pressure could not be maintained below 159/99 mmHg were withdrawn from the study. Participants were also asked to discontinue use of NSAIDs, decongestants, and over-the-counter cold and flu remedies for 2 weeks prior to study initiation.

### 2.3. Calcium and Vitamin D Washout

Enrolled participants on vitamin D therapy were required to be on a stable dose for at least 2 months prior to study initiation. All calcium supplements were discontinued for the duration of the study, and study participants received a standardized calcium supplementation as part of the study diet (below).

### 2.4. Dietary Control

All participants were placed on a liberal sodium diet for 5 days prior to their study visit to ensure standardization of sodium balance, which can dramatically influence RAAS activity. This diet consisted of their usual ad lib diet, supplemented with 150 mEq of sodium per day. Dietary potassium was also supplemented during the study diet weeks, given its crucial role in RAAS regulation, with 50 mEq of potassium chloride daily. Participants without P-HPT were given daily dietary calcium supplementation (1000 mg of calcium carbonate) to ensure similar intake between participants; participants with P-HPT did not receive any dietary calcium supplementation during the study.

### 2.5. Study Visits

**Figure 1:**
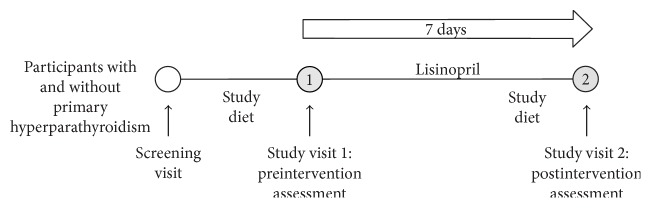
Study schema.

The study schematic is demonstrated in Figure 1. After 5 days of standardized dietary intake as outlined above, participants arrived to the outpatient research center at 8 am for study visit 1. They completed a 24-hour urine collection for creatinine, sodium, calcium, phosphate, and aldosterone, ending just prior to their arrival at the outpatient center. Participants arrived fasting overnight except for water and were instructed to lie supine for 1 hour to control for postural effects of the RAAS. Blood pressure was measured every 10 minutes with a Dinamap Pro Monitor (GE Medical). After 1 hour of supine posture, blood was drawn to measure PTH, calcium (total and ionized), plasma renin activity (PRA), and aldosterone. Participants were then discharged home with a 7-day supply of lisinopril and asked to resume their typical diet, until resuming the outlined dietary protocol 5 days prior to study visit 2. The lisinopril dose was increased to a tolerated maximum as dictated by blood pressure (see Lisinopril Dosing Protocol below).

After 7 days of lisinopril therapy, participants returned, having fasted overnight, to the outpatient research center at 8 am to repeat all study procedures conducted at baseline (study visit 2) (Figure 1). Upon study completion, participants resumed all medications that they were taking prior to study initiation.

### 2.6. Lisinopril Dosing Protocol

The goal of the intervention was to treat each participant with the maximum dose of lisinopril that could be tolerated without development of hypotension. Lisinopril was dosed twice daily to ensure that the pharmacologic effect was sustained over 24 hours. All participants were required to monitor their blood pressure at home while on lisinopril with a home sphygmomanometer for safety and titration purposes. Home blood pressure readings were reported daily to study staff. Lisinopril dosing was adjusted based on home blood pressure readings, described in Supplemental Figure 1 available online at https://doi.org/10.1155/2017/4138783.

Study staff spoke with participants by phone or electronic mail every 1-2 days during the 7-day lisinopril intervention to ensure they were compliant with the medication dosing regimen and to evaluate blood pressure readings and other symptoms that may be suggestive of adverse effects.

### 2.7. Laboratory Measurements

PTH (Beckman Coulter, Fullerton Ca), 25OHD (DiaSorin Inc., Stillwater, MN), plasma renin activity (DiaSorin, Stillwater, MN) and serum aldosterone (Siemens, Los Angeles, CA), serum and urinary electrolytes (including total and ionized calcium and phosphate), and urinary aldosterone excretion were measured at each study visit.

### 2.8. Statistical Analysis

Mean (standard deviation (SD)) descriptive values are reported. Paired *t*-tests were used to compare the main outcome variable, PTH, before and after intervention with lisinopril. Paired *t*-tests were also used to compare other biochemical and hemodynamic parameters. The change in PTH was assessed in the full study population and then according to P-HPT status. Subgroup analyses explored whether the change in PTH by lisinopril differed among those with and without vitamin D deficiency and in those with hypercalcemic versus normocalcemic P-HPT. Data analysis was performed using SAS statistical software (SAS Institute, Cary, NC).

## 3. Results

### 3.1. Study Participants

A total of 12 participants with P-HPT and 15 without P-HPT successfully completed the study protocol (Supplemental Figure 2). Participants with P-HPT were older and had higher calcium and PTH levels, though no differences in vitamin D or creatinine levels were observed (Table 1).

### 3.2. Changes in Blood Pressure and RAAS Activity with Lisinopril Intervention

Participants with P-HPT had lisinopril titrated to a maximum daily dose of 16.9 (12.8) mg (range 2.5–30 mg), and participants without P-HPT had lisinopril titrated to a maximum dose of 11.3 (7.3) mg (range 5–40 mg), *P* = 0.17. Lisinopril significantly decreased blood pressure in both groups (Table 2). Despite high dietary sodium intake that suppressed PRA, one week of lisinopril therapy resulted in substantial increases in PRA (Table 2). Serum and urinary aldosterone levels were expectantly suppressed on the high dietary sodium intake and did not change with 1 week of maximally tolerated lisinopril.

### 3.3. The Impact of Lisinopril on PTH and Calcium Parameters

**Figure 2:**
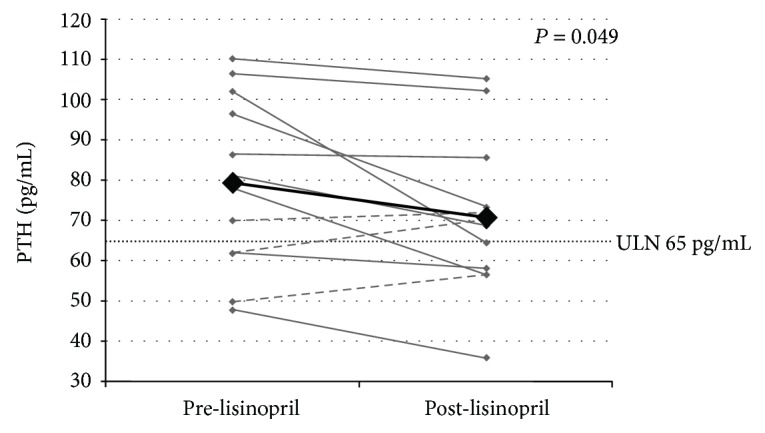
Change in PTH levels with lisinopril in primary HPT participants. Grey lines: participants with lower PTH post-lisinopril. Dashed grey lines: participants with higher PTH post-lisinopril. Black line: mean PTH. PTH, parathyroid hormone; HPT, hyperparathyroidism; ULN, upper limit of normal.

Among participants with P-HPT, there was a modest (9.5%) decrease in PTH after 1 week of lisinopril therapy (79.5 (21.6) pg/mL to 70.9 (19.6) pg/mL, ∆ = −8.6 pg/mL, *P* = 0.049, Figure 2). Of the 12 participants with P-HPT, 9/12 demonstrated a decrease in PTH following lisinopril, whereas 3/12 had an increase in PTH. Most decrements in PTH were modest (<10% of baseline) (Figure 2). Serum calcium, ionized calcium, and 24-hour urinary calcium and phosphate excretion did not significantly change with lisinopril therapy (Table 3). Among participants without P-HPT, there were no significant changes in PTH, serum calcium, ionized calcium, or 24-hour urinary calcium after lisinopril therapy (Table 3).

### 3.4. Subgroup Analyses

We assessed the influence of lisinopril on PTH in “normocalcemic” P-HPT versus “hypercalcemic” P-HPT and observed no differences (Supplemental Table 1). Similarly, there was no apparent difference in PTH changes with lisinopril among participants with low versus high 25-hydroxyvitamin D levels (Supplemental Table 2), although sample sizes were small.

## 4. Discussion

In this single-arm pilot study examining the effect of ACE inhibition on PTH levels in normal and primary hyperparathyroidism participants, we found that one week of lisinopril therapy titrated to maximally tolerated blood pressure lowering resulted in a modest and marginally statistically significant lowering of PTH levels among participants with P-HPT without any detectable change in calcium. Although lisinopril therapy similarly lowered blood pressure and raised renin activity in participants without P-HPT, there was no change in PTH or calcium levels detected. Given the many prior studies suggesting a RAAS-PTH interaction that may potentially be modifiable and clinically meaningful [11, 15, 16, 18, 25], our current findings, in addition to another recently reported study [21], suggest that short-term therapy (1–8 weeks) with RAAS inhibitors (ACE inhibitors and MR antagonists) are unlikely to induce a robust and clinically meaningful reduction in PTH in patients with P-HPT. Although our study did not assess whether a small and sustained lowering of PTH by ACE inhibitors over many years could impart benefit, this is a worthy consideration given the accruing association between PTH and cardiovascular and skeletal outcomes.

Numerous observational studies have reported an association between elevated PTH levels and cardiovascular disease [7, 10, 26, 27], which may be due to an interaction between PTH and calcium regulation and the RAAS [12, 13]. Studies investigating effects of parathyroidectomy in P-HPT have repeatedly demonstrated improvements in cardiovascular function [28–31] and decreases in RAAS activity [32, 33]. Our prior results among individuals *without* P-HPT suggested that the hypothesized interaction between PTH and the RAAS may be modifiable; the *chronic* use of ACE inhibitors and angiotensin receptor blockers is associated with lower PTH levels in a large cross-sectional study [18], and the administration of a single dose of captopril 25 mg has been shown to lower PTH levels within hours [19]. Thus, there was considerable enthusiasm to investigate whether ACE inhibition to lower RAAS activity could induce clinically meaningful PTH reductions in P-HPT.

The results of our current study are best interpreted in the context of the aforementioned prior observational data and also the recently published EPATH trial [21], a relatively large, randomized, and placebo-controlled trial that evaluated the effect of eplerenone on PTH levels in P-HPT participants. In EPATH, 110 P-HPT participants were randomized to eplerenone (up to 25–50 mg/d) versus placebo for 8 weeks. Though eplerenone induced significant reductions in blood pressure, no significant change in PTH or calcium was detected. This study has several advantages over our study, in that it had a larger sample size, longer duration of treatment, and use of placebo-control. However, a key difference was that EPATH investigated MR antagonism and not ACE inhibition as in the current study.

As previously noted, both normal and adenomatous parathyroid cells express AT1R in addition to MR [19], and prior human studies have suggested that increases in either angiotensin II and/or aldosterone may increase PTH secretion [19]. Our current study focused on the influence of ACE inhibition, which primarily lowers angiotensin II generation, and consequently aldosterone secretion. Therefore, ACE inhibitors may decrease PTH levels via decreased stimulation of *both* AT1R and MR, as opposed to MR antagonism alone, which may paradoxically increase angiotensin II generation. However, one potential limitation of lowering aldosterone secretion with ACE inhibitors is the phenomenon of aldosterone “escape,” or normalization over time [34, 35], which may or may not be a limitation of MR antagonism.

In this regard, the current study design went to great lengths to ensure that confounders of the RAAS were controlled, including dietary control of sodium and potassium, eliminating confounding medications that modulate the RAAS, and control of body posture. Lisinopril was consumed twice daily to best ensure a continuous duration of action. Further, a dose titration protocol was utilized to obtain the highest tolerated biological effect. In this context, we successfully induced blood pressure reductions and renin elevations, providing confidence that the effect of ACE inhibition was evident, yet it only induced a modest and marginally significant reduction in PTH among those with P-HPT. To some degree, these findings support our prior observations that chronic ACE inhibitor use is associated with lower PTH [18], and that a single dose of ACE inhibitor acutely lowers PTH [19]. On the other hand, the current study does not address whether a longer duration of ACE inhibitor therapy in P-HPT could have induced a sustained and durable reduction in PTH, and perhaps even in calcium. Therefore, the findings of the current study suggest that at best, ACE inhibitor use is likely to have a modest effect with unclear long-term clinical value. In this regard, it may be possible that dual blockade with both an ACE inhibitor and an MR antagonist may lead to more robust and sustained effects of PTH lowering, as both AT1R and MR would be inhibited and the potential for reversal of efficacy due to aldosterone escape with ACE inhibition would be lessened with the concurrent use of MR antagonism. However, larger clinical trials with close attention to the safety of dual blockade are needed to evaluate this further.

There are limitations to this study that are worth discussing. The most notable are the small sample size, the lack of a placebo control, and the short duration, all of which contribute to the variability of our data. Since we previously observed that ACE inhibitor mediated lowering of aldosterone and PTH [19], the current study was a natural extension of the prior. However, the current results raise the possibility that while acute ACE inhibition lowered PTH by 9.7% [19], continued ACE inhibition over the course of one week resulted in at least some re-equilibration of RAAS and PTH changes. Though we did observe a marginally significant 9.5% PTH reduction in the P-HPT group, our study was not designed to evaluate whether this PTH lowering effect would be durable over a longer period of time, or if it would result in meaningful reductions in serum calcium and cardiovascular and skeletal risk. We did not directly measure angiotensin II but instead relied on a rise in PRA to indicate ACE inhibition. And lastly, we observed no differences in aldosterone levels; however, this should not be surprising since our dietary sodium protocol suppressed levels to a near nadir. Thus, it may also be considered that while the dietary sodium standardization helped to minimize confounding of intra- and interindividual RAAS measurements, it may have also dampened the efficacy of ACE inhibition on PTH regulation.

In conclusion, this pilot intervention study demonstrated that maximally-tolerated and short-term ACE inhibitor therapy induced only modest and marginally significant decreases in PTH among participants with P-HPT, but not in participants without P-HPT. However, we caveat this conclusion by noting the limitations of our study, including the small sample size, short study duration, and lack of placebo control. Whether ACE inhibition for patients with P-HPT could induce a sustained and clinically significant reduction in PTH would require a study design with longer duration of therapy and a larger sample size.

## Supplementary Material

SUPPLEMENTAL TABLES AND FIGURES. Supplemental Table 1. Effect of baseline normocalcemic vs. hypercalcemic hyperparathyroidism on PTH with intervention. Supplemental Table 2. Effect of baseline vitamin D sufficiency vs. insufficiency on PTH with intervention. Supplemental Figure 1. Lisinopril titration protocol. Supplemental Figure 2. Consort Diagram.

## Figures and Tables

**Table 1:** Screening demographic and biochemical characteristics of study population.

	Participants without primary HPT *N* = 15	Participants with primary HPT *N* = 12
Age, years	39.6 (12.7)	51.4 (15.6)
Female, number (%)	6 (40)	5 (42)
White, number (%)	8 (53)	9 (75)
SBP, mmHg	120.0 (18.0)	121.4 (12.6)
DBP, mmHg	78.1 (12.0)	76.7 (5.2)
Number with hypertension	1	2
Number on antihypertensive therapy	0	3
PTH, pg/mL	21.8 (5.7)	94.8 (29.0)
25(OH)D, ng/mL	23.0 (8.6)	28.8 (10.7)
Serum calcium	9.6 (0.3)	11.0 (0.4)
Serum creatinine, mg/dL	0.84 (0.10)	0.83 (0.13)
Serum potassium, mmol/L	4.5 (0.5)	4.5 (0.3)

Values are mean (SD) unless otherwise noted. HPT: hyperparathyroidism; SBP: systolic blood pressure; DBP: diastolic blood pressure; PTH: parathyroid hormone; 25(OH)D: 25-hydroxyvitamin D.

**Table 2:** Blood pressure and RAAS activity before and after lisinopril intervention.

	Preintervention	Postintervention	Delta	*P* value
*With P-HPT, N* = 12				
SBP, mmHg	123.7 (15.7)	117.3 (14.7)	−6.4	0.006
DBP, mmHg	74.5 (8.7)	70.1 (7.7)	−4.4	0.003
MAP, mmHg	92.7 (12.4)	86.8 (11.1)	−5.9	0.002
Supine serum aldosterone, ng/dL	4.6 (3.0)	4.3 (3.2)	−0.3	0.50
PRA, ng/mL·hr	0.5 (0.4)	1.9 (2.7)	+1.5	0.06
Aldosterone-to-renin ratio	26.3 (39.0)	26.2 (40.5)	−0.15	0.98
24 h urinary aldosterone excretion rate, ng/dL·*μ*g/TV	11.7 (18.3)	7.8 (8.9)	−3.9	0.22
24 h urinary sodium excretion, mmol/day	239.8 (59.1)	239.6 (94.1)	−0.2	0.99
*Without P-HPT, N* = 15				
SBP, mmHg	117.4 (15.1)	108.2 (12.2)	−9.2	<0.0001
DBP, mmHg	72.1 (11.2)	66.3 (9.3)	−5.8	0.0002
MAP, mmHg	87.7 (12.6)	79.9 (9.7)	−7.8	0.003
Supine serum aldosterone, ng/dL	3.4 (1.6)	4.7 (5.0)	+1.3	0.26
PRA, ng/mL·hr	0.5 (0.4)	4.4 (6.1)	+3.9	0.02
Aldosterone-to-renin ratio	13.6 (11.7)	4.2 (5.5)	−9.4	0.0002
24 h urinary aldosterone excretion rate, ng/dL·*μ*g/TV	5.7 (3.5)	6.1 (9.1)	+0.4	0.86
24 h urinary sodium excretion, mmol/day	231.9 (82.2)	203.4 (74.9)	−28.5	0.21

Values are mean (SD) unless otherwise noted. *P* values are paired *t*-tests. RAAS: renin-angiotensin-aldosterone system; HPT: hyperparathyroidism; SBP: systolic blood pressure; DBP: diastolic blood pressure; MAP: mean arterial pressure; PRA: plasma renin activity.

**Table 3:** Markers of PTH and calcium metabolism before and after lisinopril intervention; primary HPT, without HPT, and total cohort.

	Preintervention	Postintervention	Delta	*P* value
*Primary HPT, N* = 12				
PTH, pg/mL	79.5 (21.6)	70.9 (19.6)	−8.6	0.049
Serum total calcium, mg/dL	10.6 (0.5)	10.5 (0.4)	−0.05	0.48
Ionized calcium, mmol/L	1.38 (0.06)	1.37 (0.04)	−0.009	0.51
24-hour urinary calcium excretion, mg/day	310.6 (80.8)	324.8 (100.1)	+14.2	0.51
24-hour urine phosphate excretion, mg/day	998.4 (338.0)	975.7 (454.5)	−22.7	0.84
*Without primary HPT, N* = 15				
PTH, pg/mL	33.3 (25.4)	32.5 (16.7)	−0.78	0.80
Serum total calcium, mg/dL	9.4 (0.4)	9.5 (0.3)	+0.03	0.80
Ionized calcium, mmol/L	1.21 (0.07)	1.21 (0.07)	−0	1.0
24-hour urinary calcium excretion, mg/day	171.0 (90.6)	153.2 (70.4)	−17.8	0.34
24-hour urine phosphate excretion, mg/day	681.8 (406.9)	597.9 (241.6)	−165.4	0.26
*Total cohort, N* = 27				
PTH, pg/mL	53.8 (33.0)	49.6 (26.3)	−4.2	0.10
Serum total calcium, mg/dL	9.9 (0.7)	9.9 (0.6)	−0.004	0.96
Ionized calcium, mmol/L	1.29 (0.10)	1.28 (0.10)	−0.007	0.60
24-hour urinary calcium excretion, mg/day	233.0 (110.4)	229.4 (120.2)	−3.60	0.80
24-hour urine phosphate excretion, mg/day	822.5 (404.0)	765.8 (394.2)	−56.7	0.37

Values are mean (SD) unless otherwise noted. *P* values are paired *t*-tests. HPT: hyperparathyroidism; PTH: parathyroid hormone.
