# Supplementary material for: Angiotensin-Converting Enzyme Inhibition and Parathyroid Hormone Secretion
Source: Int J Endocrinol. 2017 Jul 20;2017:4138783. doi: 10.1155/2017/4138783 (PMC5541811; doi:10.1155/2017/4138783)

SUPPLEMENTAL TABLES AND FIGURES

Supplemental Table 1. Effect of baseline normocalcemic vs. hypercalcemic hyperparathyroidism on PTH with intervention.

	Pre-intervention	Post-intervention	Delta, P-value	Two-sample T-test
**Primary HPT**				0.89
Normocalcemic PTH, pg/ml, N=4	77.6 (15.0)	68.1 (6.8)	-8.2, p=0.17	
Hypercalcemic PTH, pg/ml, N=9	82.2 (24.1)	72.2 (24.0)	-9.4, p=0.18	

Values are mean (SD) unless otherwise noted.

Normocalcemia defined as medical chart baseline Ca ≤10.7mg/dl.

PTH, parathyroid hormone; HPT, hyperparathyroidism.

Supplemental Table 2. Effect of baseline vitamin D sufficiency vs. insufficiency on PTH with intervention.

	Pre-intervention	Post-intervention	Delta, P-value	Two-sample T-test
**Total Cohort**				
PTH, pg/ml when 25OHD <20 ng/ml, n=9	64.3 (42.5)	58.4 (33.4)	-5.9, p=0.28	0.63
PTH, pg/ml when 25OHD ≥20 ng/ml, n=21	50.3 (27.1)	45.2 (27.0)	-3.4, p=0.23	
**Primary HPT**				
PTH, pg/ml when 25OHD <20 ng/ml, n=3	104.6 (7.1)	93.7 (17.6)	-10.8, p=0.22	0.76
PTH, pg/ml when 25OHD ≥20 ng/ml, n=9	71.1 (17.7)	63.3 (13.8)	-7.8, p=0.15	

Values are mean (SD) unless otherwise noted.

PTH, parathyroid hormone; HPT, hyperparathyroidism.

Supplemental Figure 1. Lisinopril titration protocol.

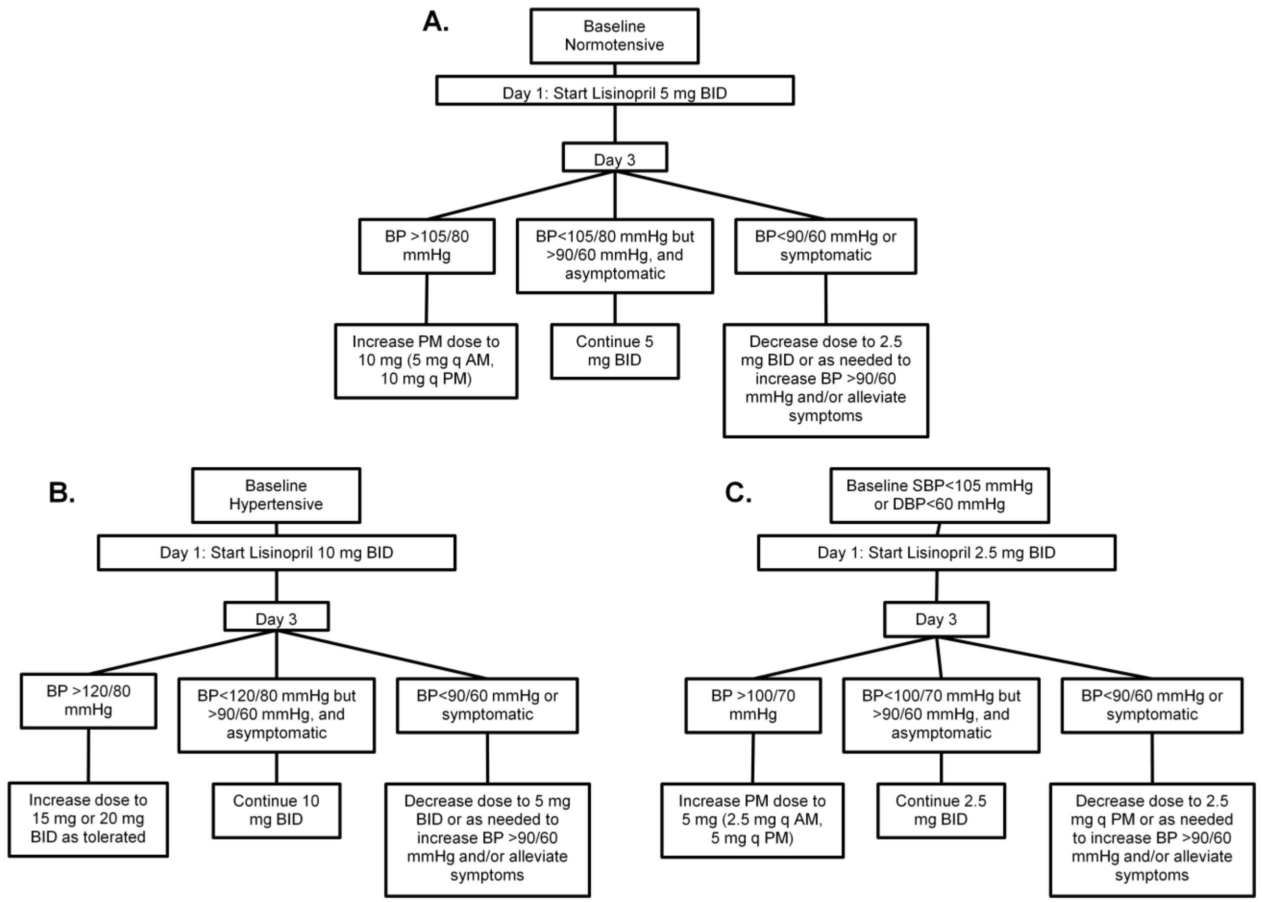

Dosing algorithm A, B, or C based on baseline blood pressure:

A. Normotensive (<140/90 mmHg) at baseline

B. Hypertensive (≥140/90 mmHg) at baseline

C. SBP <105 mmHg or DBP <60 mmHg at baseline

After initial lisinopril dose chosen based on baseline blood pressure, participants monitored their blood pressure at home with a home sphygmomanometer. Home blood pressure readings were reported to study staff and lisinopril dosing was adjusted per protocol.

BP, blood pressure; SBP, systolic blood pressure; DBP, diastolic blood pressure.

Supplemental Figure 2. Consort Diagram.

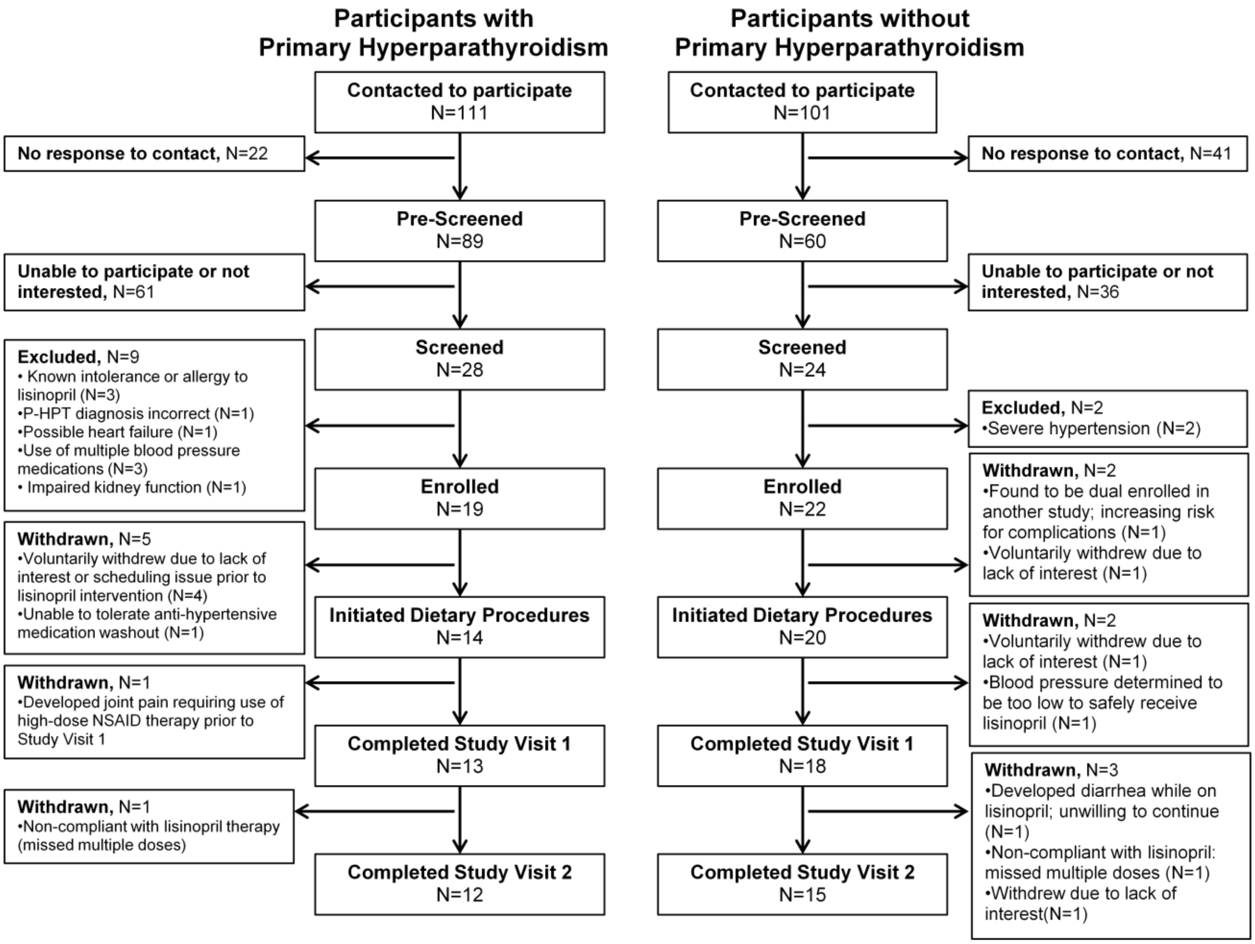

Supplement: Supplementary file 1 — SUPPLEMENTAL TABLES AND FIGURES. Supplemental Table 1. Effect of baseline normocalcemic vs. hypercalcemic hyperparathyroidism on PTH with intervention. Supplemental Table 2. Effect of baseline vitamin D sufficiency vs. insufficiency on PTH with intervention. Supplemental Figure 1. Lisinopril titration protocol. Supplemental Figure 2. Consort Diagram. [file 4138783.f1.pdf]
